# Supplementary material for: Assignment of Absolute Configuration of Bromoallenes by Vacuum-Ultraviolet Circular Dichroism (VUVCD)
Source: Molecules. 2021 Feb 27;26(5):1296. doi: 10.3390/molecules26051296 (PMC7957760; doi:10.3390/molecules26051296)
Supplement: Supplementary file 1 [file molecules-26-01296-s001.pdf]

Assignment of absolute configuration of bromoallenes by vacuum-ultraviolet circular dichroism (VUVCD)

Taiki Umezawa[1,*], Nakaba Mizutani[1], Koichi Matsuo[2], Yuugo Tokunaga[3], Fuyuhiko Matsuda[1], Tatsuo Nehira[3,4,*]

[1]Division of Environmental Materials Science, Graduate School of Environmental Science, Hokkaido University, N10W5 Sapporo 060-0810, Japan, e-mail: umezawa@ees.hokudai.ac.jp,
[2]Hiroshima Synchrotron Radiation Center (HiSOR), Hiroshima University, 2-313 Kagamiyama, Higashi-Hiroshima 739-0046, Japan
[3]Faculty of Integrated Arts and Sciences, Hiroshima University, 1-7-1 Kagamiyama, Higashi-Hiroshima, 739-8521, Japan
[4]Graduate School of Integrated Sciences for Life, Hiroshima University, 1-7-1 Kagamiyama, Higashi-Hiroshima, 739-8521, Japan, e-mail: tnehira@hiroshima-u.ac.jp

Table of Contents

Assignment of Absolute Configuration of Propargyl Alcohols 6 and 7 1

NMR Spectra 4

Assignment of Absolute Configuration of Propargyl Alcohols 6 and 7

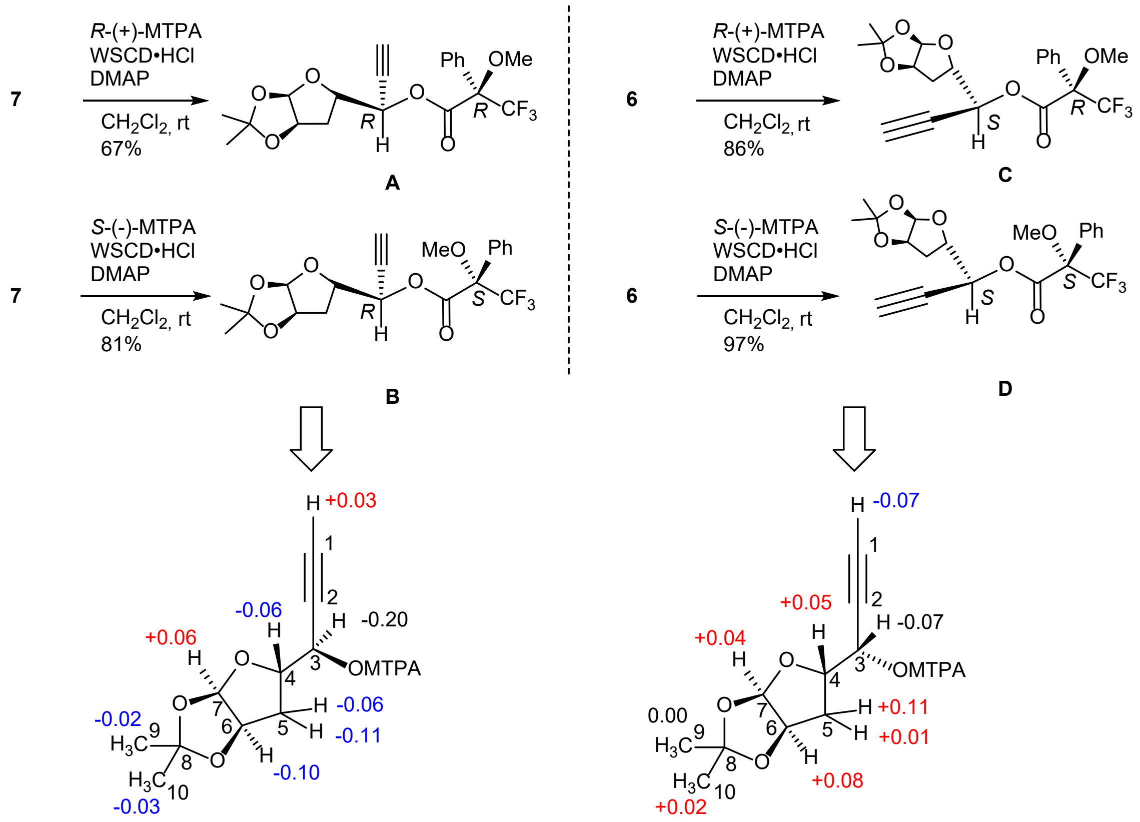

position	δ^R (**A**) [ppm]	δ^S (**B**) [ppm]	$\Delta\delta = \delta^S - \delta^R$	δ^R (**C**) [ppm]	δ^S (**D**) [ppm]	$\Delta\delta = \delta^S - \delta^R$
9 (CH_3)	1.32	1.30	−0.02	1.31	1.31	0
10 (CH_3)	1.52	1.49	−0.03	1.47	1.49	+0.02
5a (CH_2)	1.96	1.85	−0.11	1.81	1.82	+0.01
5b (CH_2)	2.24	2.18	−0.06	2.14	2.25	+0.11
1 (CH)	2.53	2.56	+0.03	2.58	2.51	−0.07
4 (CH)	4.55	4.49	−0.06	4.42	4.47	+0.05
6 (CH)	4.69	4.59	−0.10	4.67	4.75	+0.08
3 (CH)	5.72	5.52	−0.20	5.67	5.60	−0.07
7 (CH)	5.83	5.89	+0.06	5.80	5.84	+0.04

Figure S1

MTPA Ester **A** To a solution of Propargyl Alcohol **7** (6.00 mg, 0.0303 mmol) in CH_2Cl_2 (150 μL) were added *R*-*α*-Methoxy-*α*-(trifluoromethyl) phenylacetic Acid (10.5 mg, 0.0450 mmol), DMAP (0.400 mg, 3.00 μmol) and EDCI (11.5 mg, 0.0600 mmol) at room temperature under Ar atmosphere. The mixture was stirred at room temperature for 20 min, quenched with saturated $NaHCO_3$, extracted with AcOEt, washed with brine, dried over Na_2SO_4, filtered, and concentrated in *vacuo*. The crude product was purified by silica gel column chromatography (AcOEt:hexane = 5:95) to give the MTPA ester **A** (10.7 mg, 0.0258 mmol, 86%) as a colorless oil: ^{1}H NMR (400 MHz, $CDCl_3$) δ 1.32 (3H, s), 1.52 (3H, s), 1.96 (1H, ddd, *J* = 14.0, 10.3, 4.9 Hz), 2.24 (1H, dd, *J* = 13.7, 4.9 Hz), 2.53 (1H, d, *J* = 2.0 Hz), 3.54 (3H, s), 4.55 (1H, ddd, *J* = 10.3, 5.1, 2.9 Hz), 4.69 (1H, t, *J* = 4.1 Hz), 5.72 (1H, d, *J* = 3.4 Hz), 5.83 (1H, t, *J* = 2.7 Hz), 7.38 - 7.45 (3H, m), 7.52 - 7.54 (2H, m)

MTPA Ester **B** To a solution of Propargyl Alcohol **7** (5.60 mg, 0.0280 mmol) in CH_2Cl_2 (140 μL) were added *S*-*α*-Methoxy-*α*-(trifluoromethyl)phenylacetic Acid (9.80, 0.0420 mmol), DMAP (0.340 mg, 2.80 μmol) and EDCI (10.7 mg, 0.0560 mmol) at room temperature under Ar atmosphere. The mixture was stirred at room temperature for 20 min, quenched with saturated $NaHCO_3$, extracted with AcOEt, washed with brine, dried over Na_2SO_4, filtered, and concentrated in *vacuo*. The crude product was purified by silica gel column chromatography (AcOEt:hexane = 5:95) to give the MTPA ester **B** (11.2 mg, 0.0270 mmol, 97%) as a colorless oil: ^{1}H NMR (400 MHz, $CDCl_3$) δ 1.25 (3H, s), 1.49 (3H, s), 1.84 (1H, ddd, *J* = 13.8, 10.2, 4.4 Hz), 2.16 (1H, dd, *J* = 13.7, 4.9 Hz), 2.57 (1H, d, *J* = 2.4 Hz), 3.63 (3H, s), 4.49 (1H, ddd, *J* = 10.3, 4.9, 3.4 Hz), 4.58 (1H, t, *J* = 4.1 Hz), 5.50 (1H, d, *J* = 3.4 Hz), 5.89 (1H, t, *J* = 2.7 Hz), 7.36 - 7.42 (3H, m), 7.52 - 7.55 (2H, m)

MTPA Ester **C** To a solution of Propargyl Alcohol **6** (5.80 mg, 0.0240 mmol) in CH_2Cl_2 (0.5 mL) were added *R*-*α*-Methoxy-*α*-(trifluoromethyl)phenylacetic Acid (16.9 mg, 0.0720 mmol), DMAP (0.290 mg, 2.40 μmol) and EDCI (16.1 mg, 0.0840 mmol) at room temperature under Ar atmosphere. The mixture was stirred at room temperature for 20 min, quenched with saturated $NaHCO_3$, extracted with AcOEt, washed with brine, dried over Na_2SO_4, filtered, and concentrated in *vacuo*. The crude product was purified by silica gel column chromatography (AcOEt:hexane = 5:95) to give the MTPA ester **C** (6.70 mg, 0.0160 mmol, 67%) as a colorless oil: ^{1}H NMR (400 MHz, $CDCl_3$) δ 1.32 (3H, s), 1.47 (3H, s), 1.80 (1H, ddd, *J* = 14.4, 9.5, 4.9 Hz), 2.13 (1H, dd, *J* = 13.4, 5.4 Hz), 2.58 (1H, d, *J* = 2.0 Hz), 3.58 (3H, s), 4.42 (1H, qt, *J* = 5.2 Hz), 4.67 (1H, t, *J* = 4.1 Hz), 5.67 (1H, dd, *J* = 5.6, 2.0 Hz), 5.80 (1H, d, *J* = 3.4 Hz), 7.38 - 7.41 (3H, m), 7.54 - 7.57 (2H, m)

MTPA Ester **D** To a solution of Propargyl Alcohol **6** (5.20 mg, 0.0260 mmol) in CH_2Cl_2 (0.5 mL) were added *S*-*α*-Methoxy-*α*-(trifluoromethyl)phenylacetic Acid (18.3 mg, 0.0780 mmol), DMAP (0.320 mg, 2.60 μmol) and EDCI (17.4 mg, 0.0910 mmol) at room temperature under Ar atmosphere. The mixture was stirred at room temperature for 20 min, quenched with saturated $NaHCO_3$, extracted with AcOEt, washed with brine, dried over Na_2SO_4, filtered, and concentrated in *vacuo*. The crude product was purified by silica gel column chromatography (AcOEt:hexane = 5:95) to give the MTPA ester **D** (8.70 mg, 0.0210 mmol, 81%) as a colorless oil: ^{1}H NMR (400 MHz, $CDCl_3$) δ 1.25 (3H, s), 1.49 (3H, s), 1.82 (1H, ddd, *J* = 14.6, 9.8, 4.9 Hz), 2.25 (1H, dd, *J* = 13.7, 4.9 Hz), 2.51 (1H, d, *J* = 2.4 Hz), 3.59 (3H, s), 4.47 (1H, ddd, *J* = 11.3, 6.6, 4.9 Hz), 4.75 (1H, t, *J* = 4.1 Hz), 5.60 (1H, dd, *J* = 7.1, 2.0 Hz), 5.84 (1H, d, *J* = 3.9 Hz), 7.29 - 7.42 (3H, m), 7.54 - 7.56 (2H, m)

NMR Spectra

6

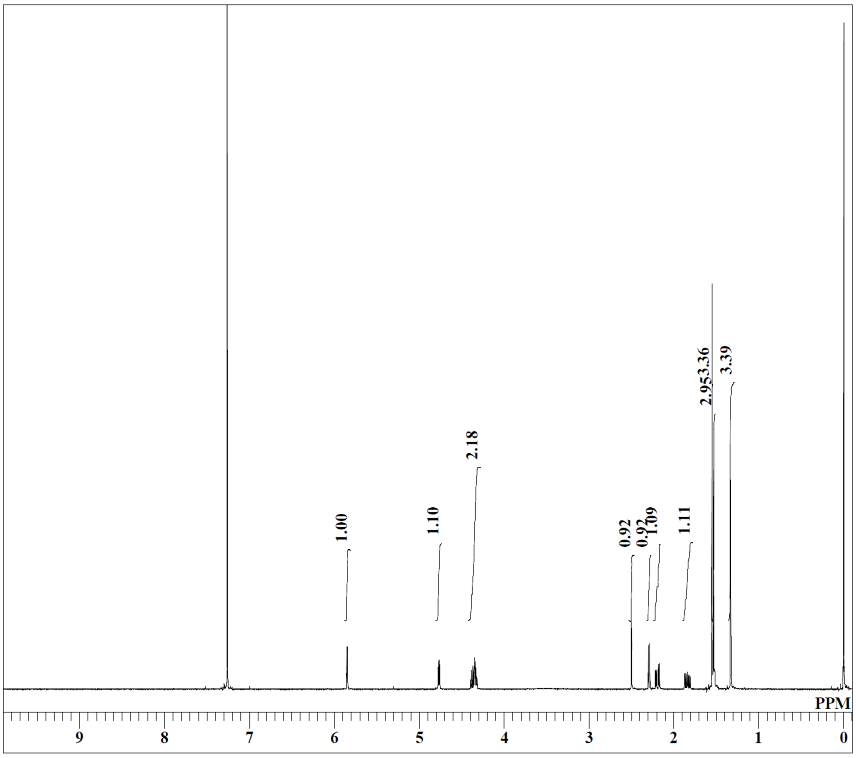

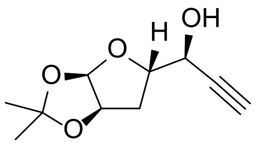

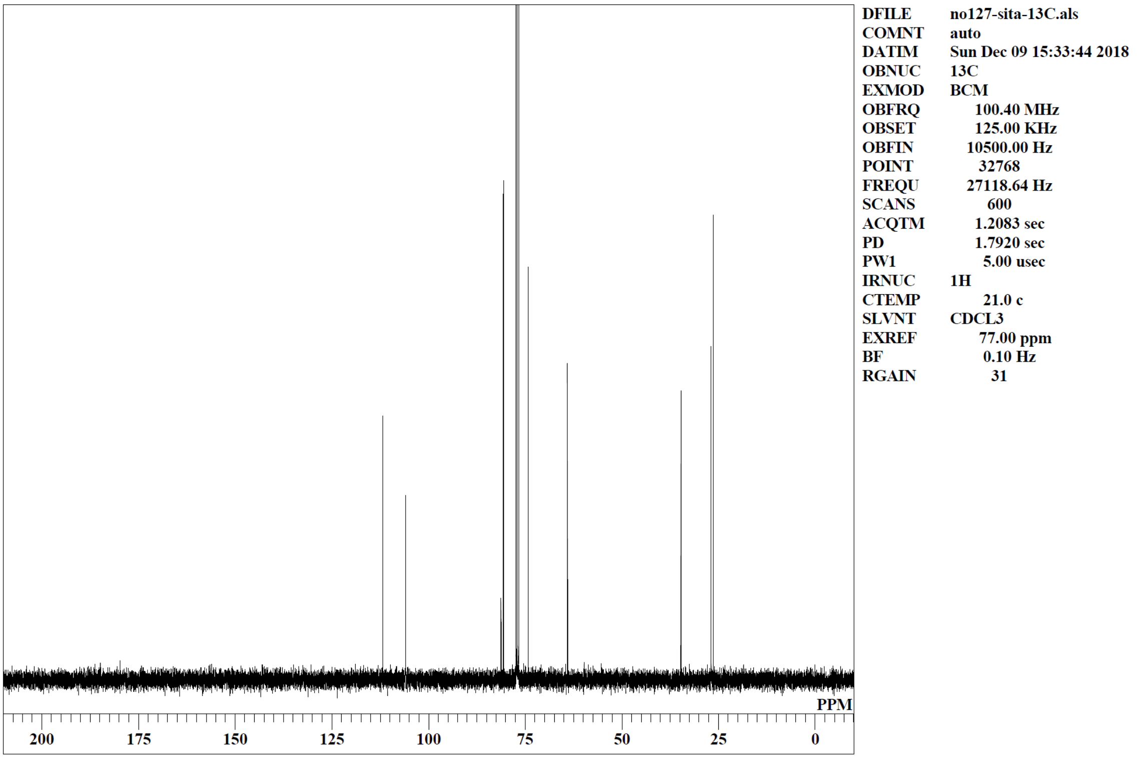

7

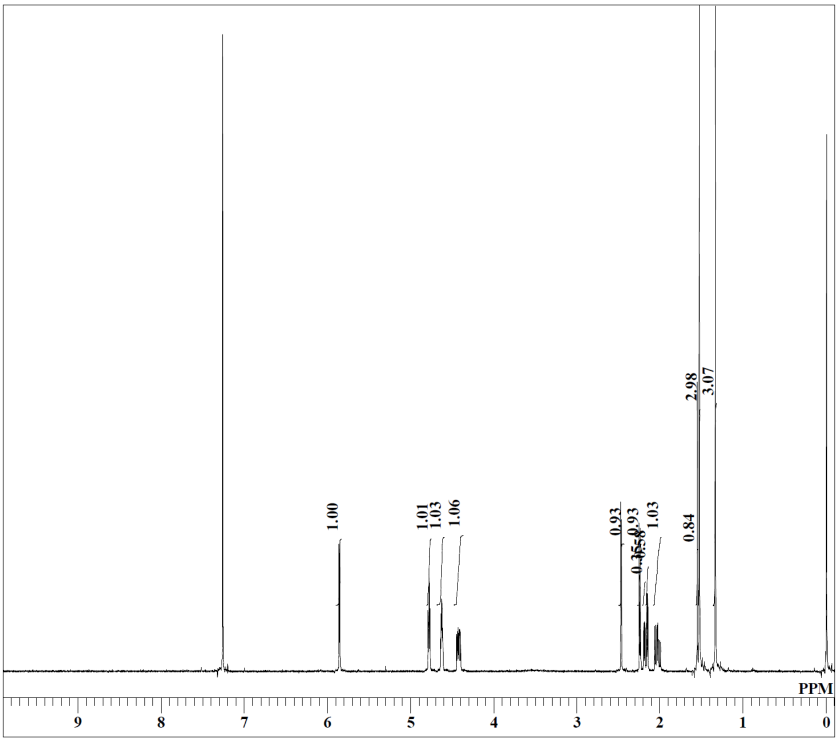

DFILE	no126-recrystalization-sol.als
COMNT	
DATIM	Sun Feb 25 18:19:32 2018
OBNUC	1H
EXMOD	NON
OBFRQ	399.65 MHz
OBSET	124.00 KHz
OBFIN	10500.00 Hz
POINT	16384
FREQU	7992.01 Hz
SCANS	8
ACQTM	2.0500 sec
PD	4.9500 sec
PW1	6.00 usec
IRNUC	1H
CTEMP	22.1 c
SLVNT	CDCL3
EXREF	7.26 ppm
BF	0.10 Hz
RGAIN	25

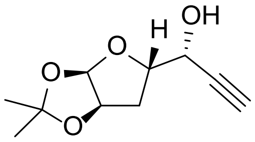

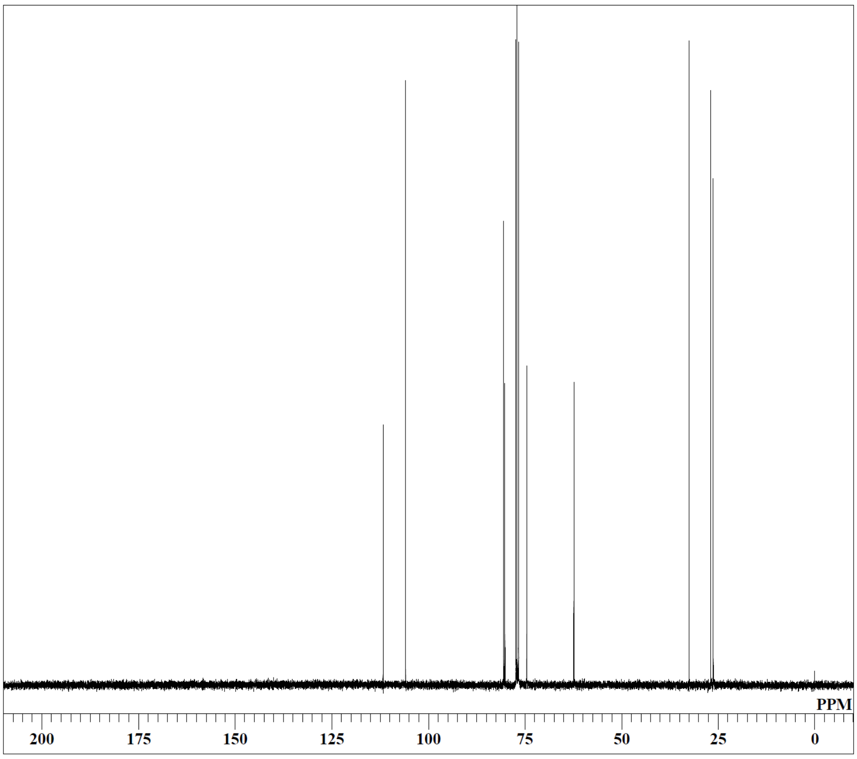

DFILE	no126-ue-13C.als
COMNT	auto
DATIM	Sun Feb 25 21:54:01 2018
OBNUC	13C
EXMOD	BCM
OBFRQ	100.40 MHz
OBSET	125.00 KHz
OBFIN	10500.00 Hz
POINT	32768
FREQU	27118.64 Hz
SCANS	3600
ACQTM	1.2083 sec
PD	1.7920 sec
PW1	5.00 usec
IRNUC	1H
CTEMP	20.5 c
SLVNT	CDCL3
EXREF	77.00 ppm
BF	0.10 Hz
RGAIN	32

10

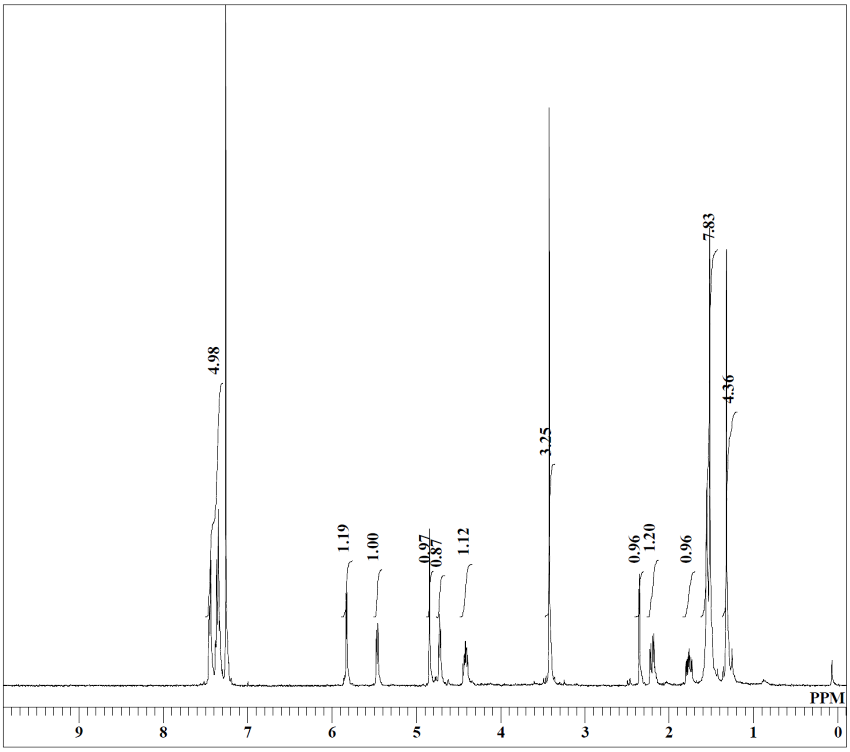

DFILE no30-sita.als
COMNT
DATIM Mon Jun 05 19:50:48 2017
OBNUC 1H
EXMOD NON
OBFRQ 399.65 MHz
OBSET 124.00 KHz
OBFIN 10500.00 Hz
POINT 16384
FREQU 7992.01 Hz
SCANS 8
ACQTM 2.0500 sec
PD 4.9500 sec
PW1 6.60 usec
IRNUC 1H
CTEMP 23.7 c
SLVNT CDCL3
EXREF 7.26 ppm
BF 1.00 Hz
RGAIN 25

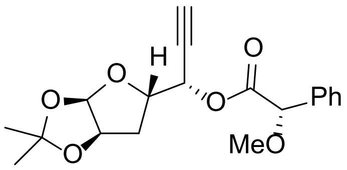

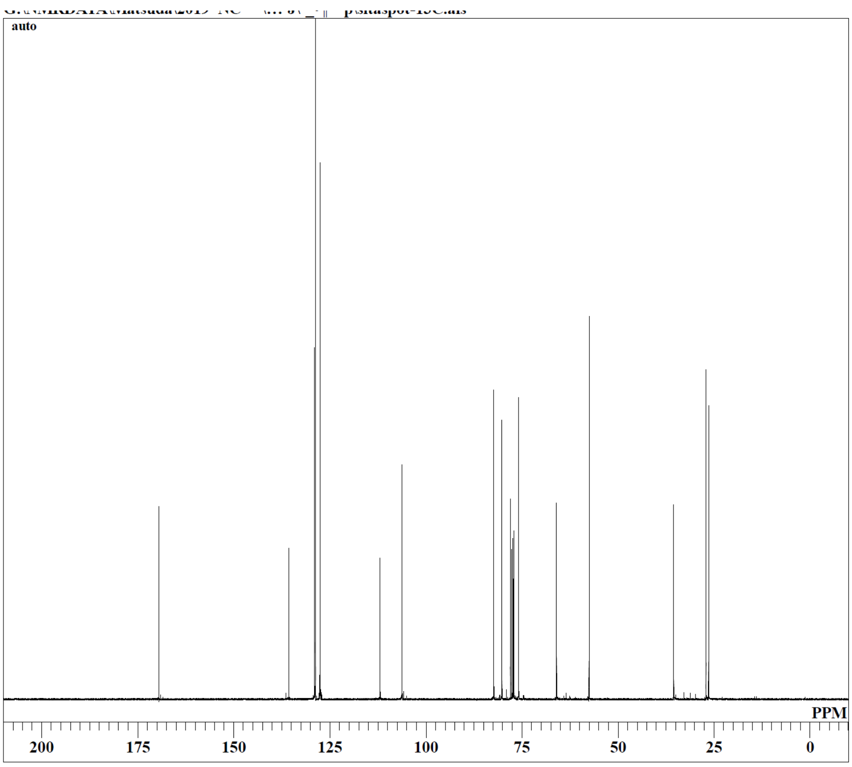

DFILE sitaspot-13C.als
COMNT auto
DATIM Thu Jun 15 22:59:48 2017
OBNUC 13C
EXMOD BCM
OBFRQ 100.40 MHz
OBSET 125.00 KHz
OBFIN 10500.00 Hz
POINT 32768
FREQU 27118.64 Hz
SCANS 3000
ACQTM 1.2083 sec
PD 1.7920 sec
PW1 4.80 usec
IRNUC 1H
CTEMP 22.7 c
SLVNT CDCL3
EXREF 77.00 ppm
BF 0.12 Hz
RGAIN 31

9

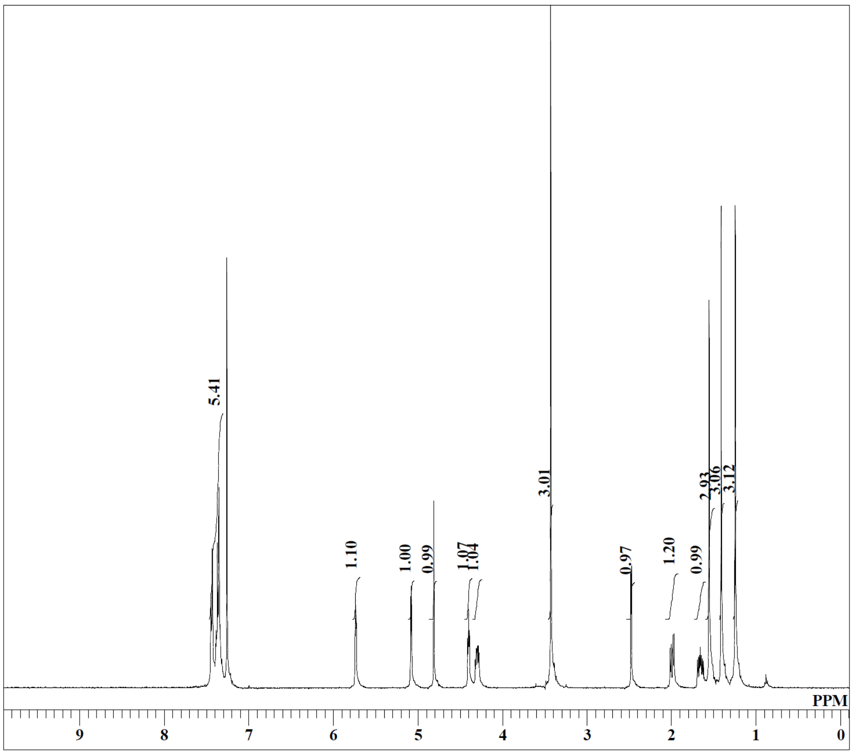

DFILE	no33-ue.als
COMNT	
DATIM	Mon Jun 05 19:58:03 2017
OBNUC	1H
EXMOD	NON
OBFRQ	399.65 MHz
OBSET	124.00 KHz
OBFIN	10500.00 Hz
POINT	16384
FREQU	7992.01 Hz
SCANS	8
ACQTM	2.0500 sec
PD	4.9500 sec
PW1	6.60 usec
IRNUC	1H
CTEMP	22.9 c
SLVNT	CDCL3
EXREF	7.26 ppm
BF	1.00 Hz
RGAIN	24

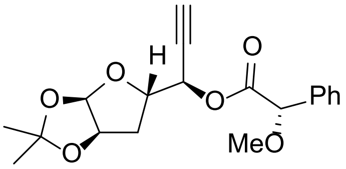

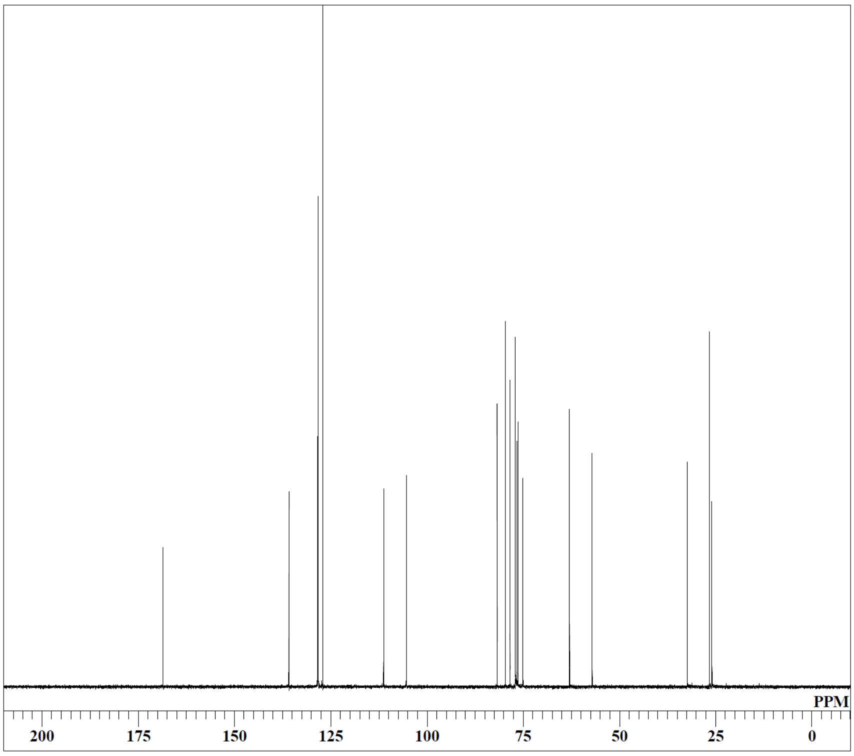

DFILE	no33-ue-13C2.als
COMNT	auto
DATIM	Thu Jun 08 23:05:28 2017
OBNUC	13C
EXMOD	BCM
OBFRQ	100.40 MHz
OBSET	125.00 KHz
OBFIN	10500.00 Hz
POINT	32768
FREQU	27118.64 Hz
SCANS	3600
ACQTM	1.2083 sec
PD	1.7920 sec
PW1	4.80 usec
IRNUC	1H
CTEMP	22.6 c
SLVNT	CDCL3
EXREF	77.00 ppm
BF	0.12 Hz
RGAIN	31

11

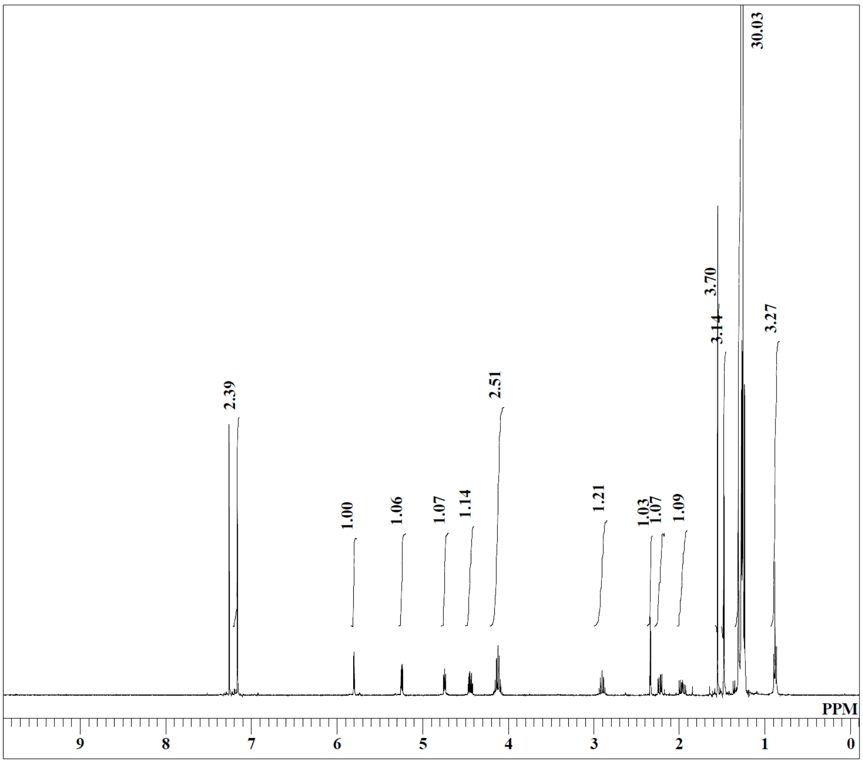

DFILE	no63-2-column1.als
COMNT	
DATIM	Fri Sep 08 14:55:12 2017
OBNUC	1H
EXMOD	NON
OBFRQ	399.65 MHz
OBSET	124.00 KHz
OBFIN	10500.00 Hz
POINT	16384
FREQU	7992.01 Hz
SCANS	8
ACQTM	2.0500 sec
PD	4.9500 sec
PW1	6.00 usec
IRNUC	1H
CTEMP	24.3 c
SLVNT	CDCL3
EXREF	7.26 ppm
BF	0.10 Hz
RGAIN	21

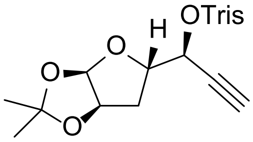

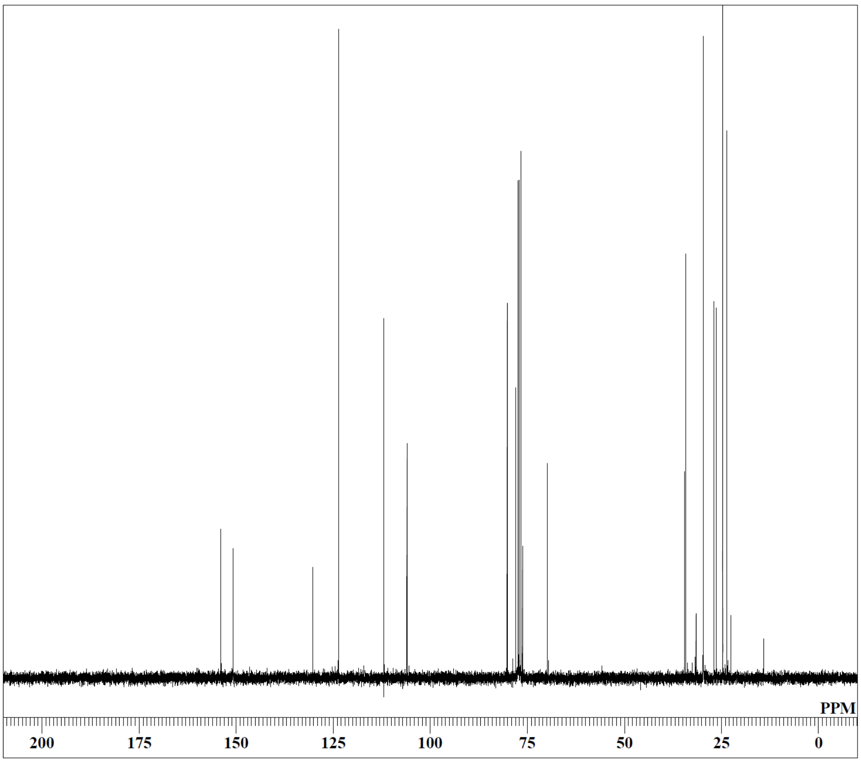

DFILE	no63-13C.als
COMNT	auto
DATIM	Sat Sep 09 18:01:24 2017
OBNUC	13C
EXMOD	BCM
OBFRQ	100.40 MHz
OBSET	125.00 KHz
OBFIN	10500.00 Hz
POINT	32768
FREQU	27118.64 Hz
SCANS	600
ACQTM	1.2083 sec
PD	1.7920 sec
PW1	5.00 usec
IRNUC	1H
CTEMP	23.9 c
SLVNT	CDCL3
EXREF	77.00 ppm
BF	0.10 Hz
RGAIN	31

(1*S*)-12

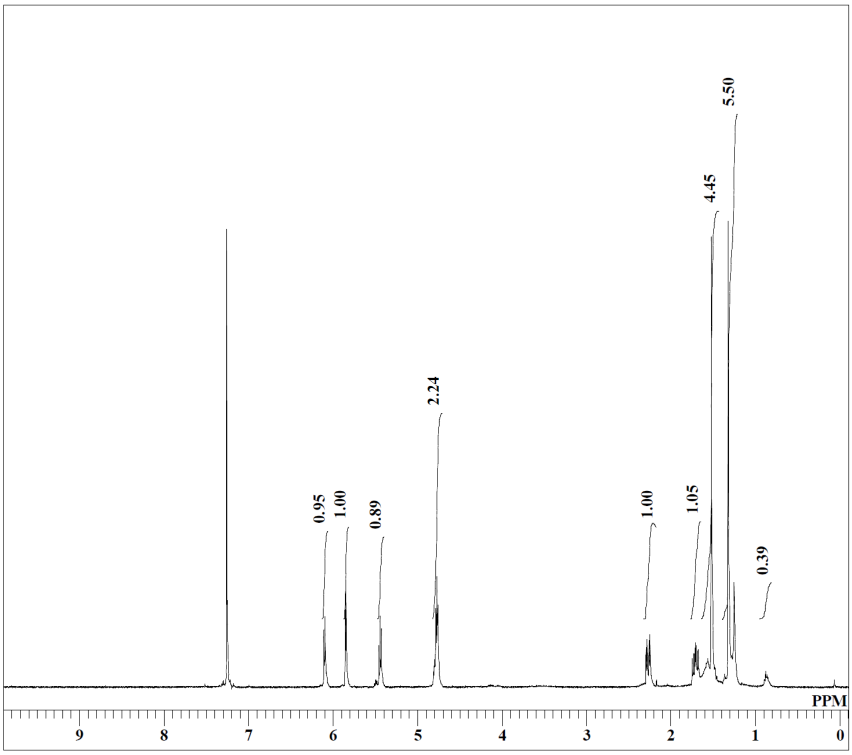

DFILE	no95-1H_study.als
COMNT	No.66 THF column C
DATIM	Thu Nov 16 20:06:35 2017
OBNUC	1H
EXMOD	NON
OBFRQ	399.65 MHz
OBSET	124.00 KHz
OBFIN	10500.00 Hz
POINT	16384
FREQU	7992.01 Hz
SCANS	16
ACQTM	2.0500 sec
PD	4.9500 sec
PW1	6.00 usec
IRNUC	1H
CTEMP	23.3 c
SLVNT	CDCL3
EXREF	7.26 ppm
BF	0.10 Hz
RGAIN	24

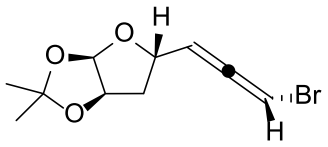

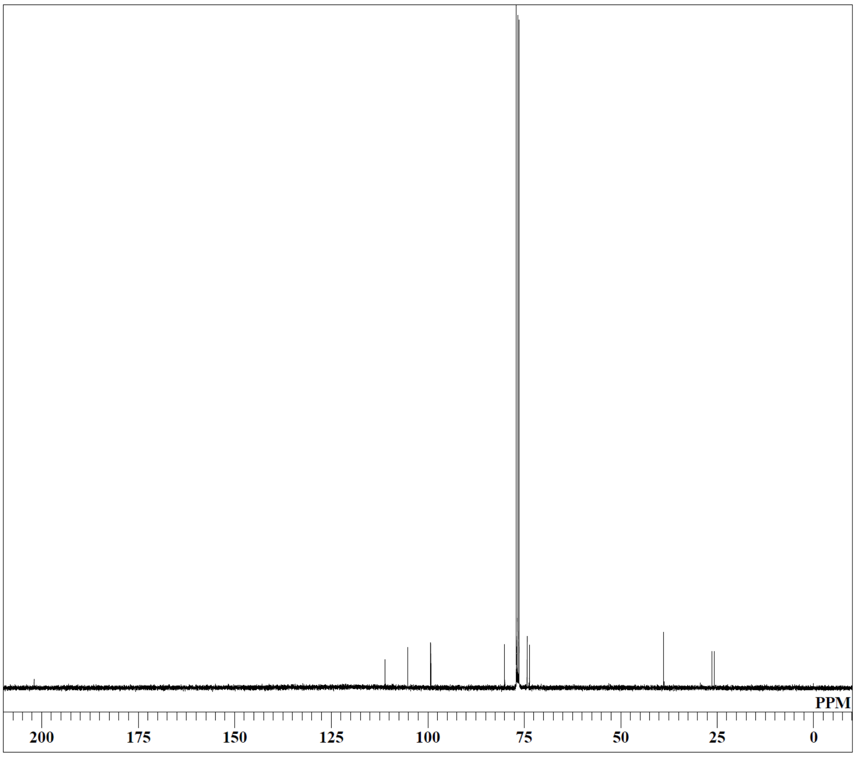

DFILE	no95-13C.als
COMNT	auto
DATIM	Fri Nov 17 08:12:39 2017
OBNUC	13C
EXMOD	BCM
OBFRQ	100.40 MHz
OBSET	125.00 KHz
OBFIN	10500.00 Hz
POINT	32768
FREQU	27118.64 Hz
SCANS	14400
ACQTM	1.2083 sec
PD	1.7920 sec
PW1	5.00 usec
IRNUC	1H
CTEMP	22.2 c
SLVNT	CDCL3
EXREF	77.00 ppm
BF	0.10 Hz
RGAIN	32

(1*S*)-13

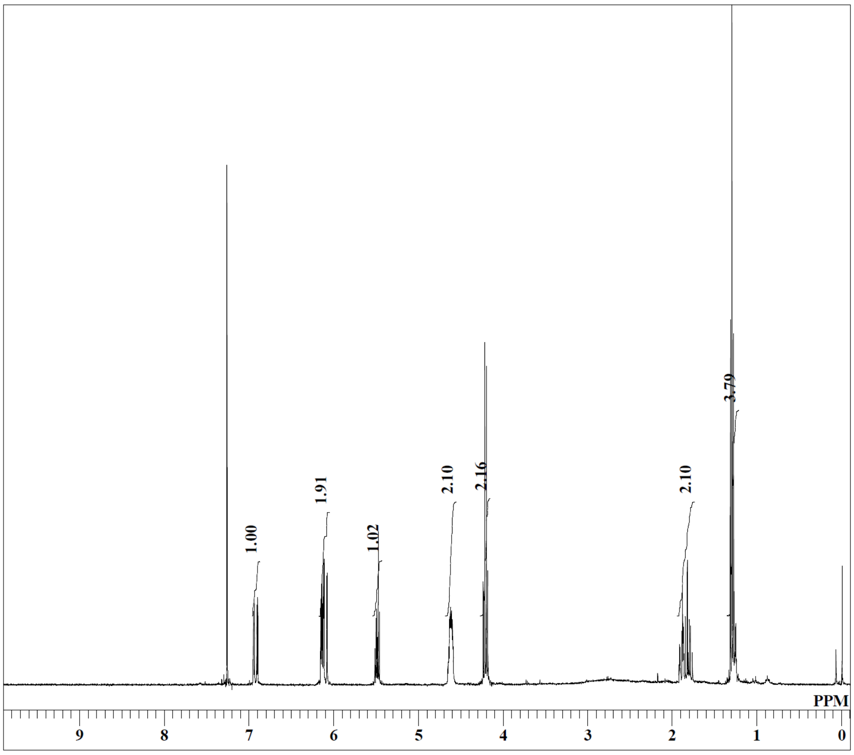

DFILE no3-sita-teacher.als
COMNT No.66 THF column C
DATIM Thu Jan 11 20:08:53 2018
OBNUC 1H
EXMOD NON
OBFRQ 399.65 MHz
OBSET 124.00 KHz
OBFIN 10500.00 Hz
POINT 16384
FREQU 7992.01 Hz
SCANS 4
ACQTM 2.0500 sec
PD 4.9500 sec
PW1 6.00 usec
IRNUC 1H
CTEMP 22.0 c
SLVNT CDCL3
EXREF 7.26 ppm
BF 0.10 Hz
RGAIN 23

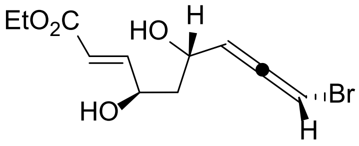

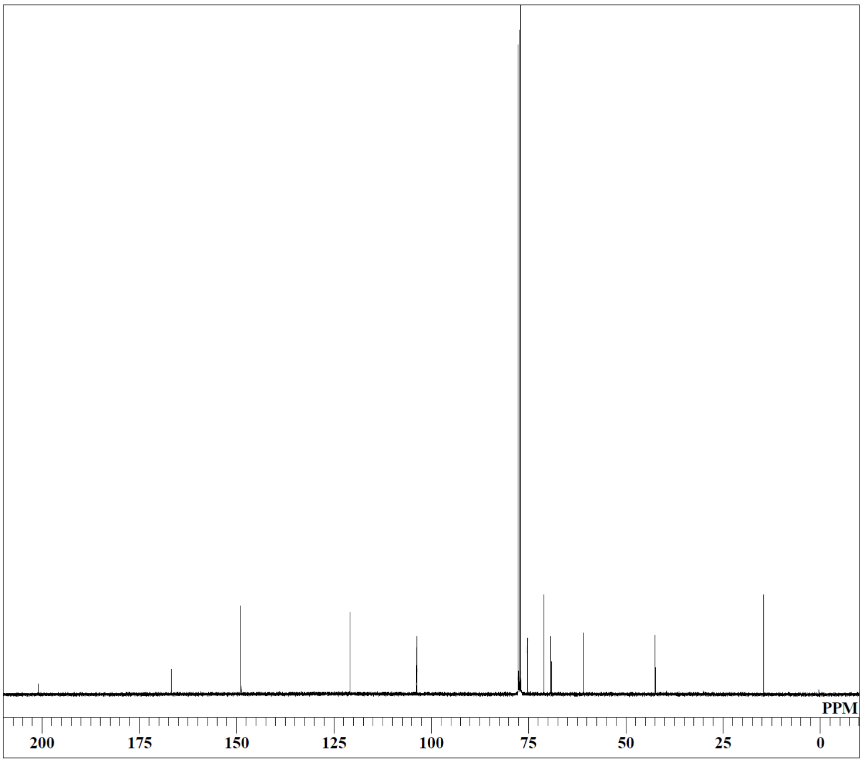

DFILE no3-sita-13C.als
COMNT auto
DATIM Fri Jan 12 07:52:17 2018
OBNUC 13C
EXMOD BCM
OBFRQ 100.40 MHz
OBSET 125.00 KHz
OBFIN 10500.00 Hz
POINT 32768
FREQU 27118.64 Hz
SCANS 14000
ACQTM 1.2083 sec
PD 1.7920 sec
PW1 5.00 usec
IRNUC 1H
CTEMP 21.0 c
SLVNT CDCL3
EXREF 77.00 ppm
BF 0.10 Hz
RGAIN 31

(1*S*)-14

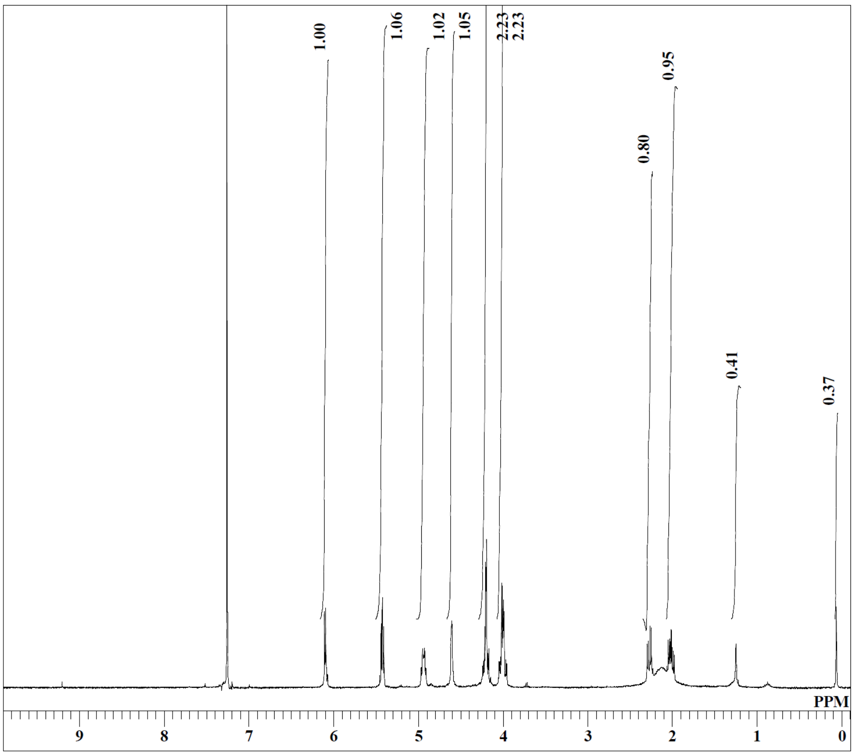

DFILE no98-2-1H.study.als
COMNT No.66 THF column C
DATIM Sat Nov 25 19:35:02 2017
OBNUC 1H
EXMOD NON
OBFRQ 399.65 MHz
OBSET 124.00 KHz
OBFIN 10500.00 Hz
POINT 16384
FREQU 7992.01 Hz
SCANS 8
ACQTM 2.0500 sec
PD 4.9500 sec
PW1 6.00 usec
IRNUC 1H
CTEMP 20.3 c
SLVNT CDCL3
EXREF 7.26 ppm
BF 0.10 Hz
RGAIN 22

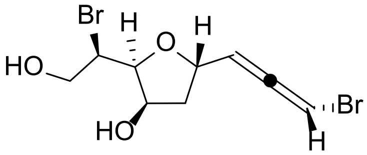

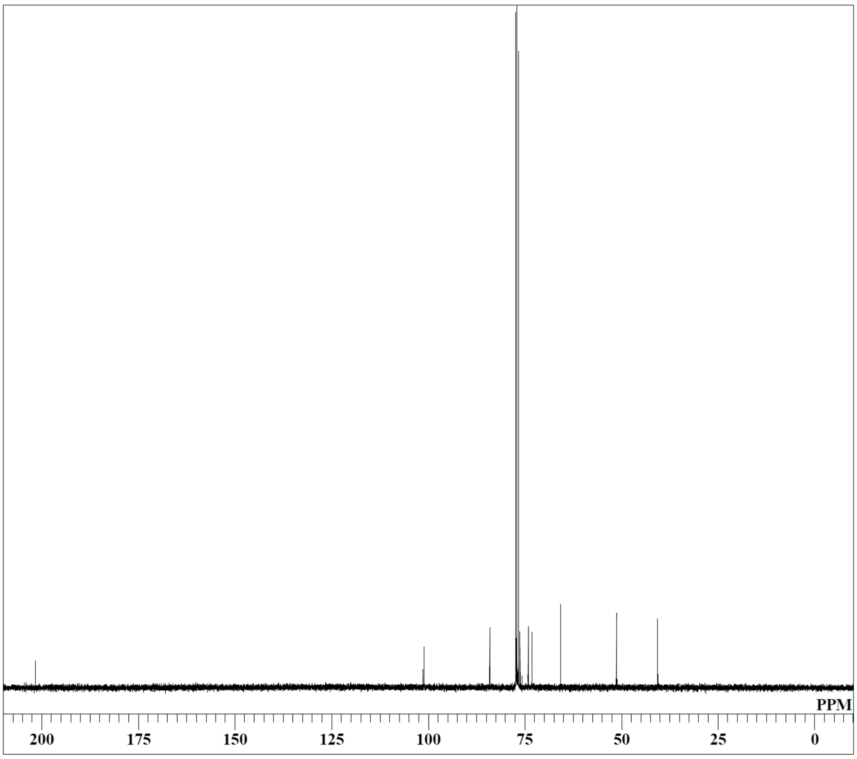

DFILE no98-2-13C.als
COMNT auto
DATIM Sat Nov 25 23:21:41 2017
OBNUC 13C
EXMOD BCM
OBFRQ 100.40 MHz
OBSET 125.00 KHz
OBFIN 10500.00 Hz
POINT 32768
FREQU 27118.64 Hz
SCANS 4480
ACQTM 1.2083 sec
PD 1.7920 sec
PW1 5.00 usec
IRNUC 1H
CTEMP 19.2 c
SLVNT CDCL3
EXREF 77.00 ppm
BF 0.10 Hz
RGAIN 31

(1*S*)-15

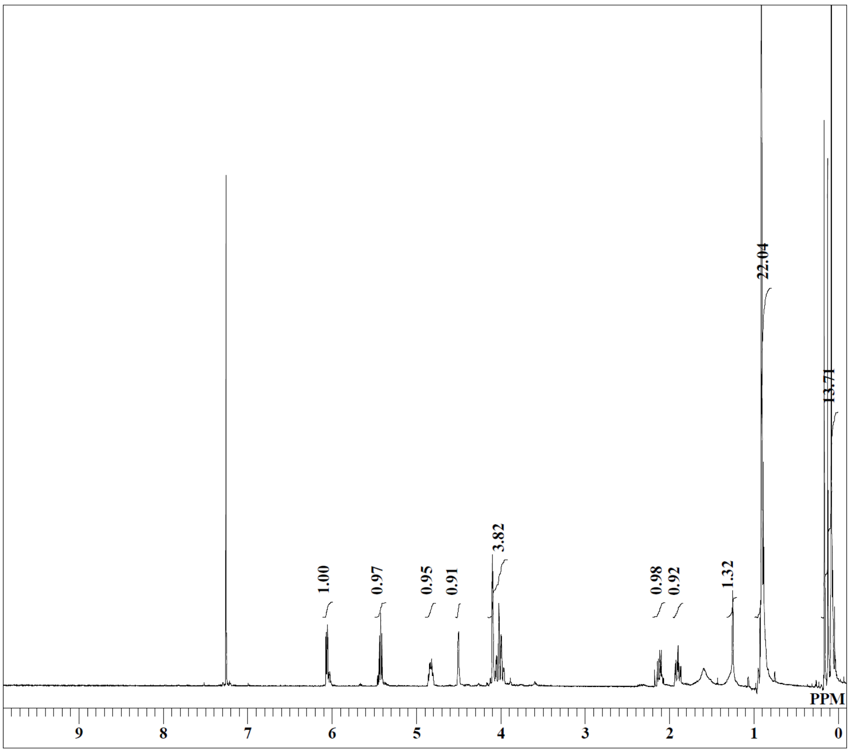

DFILE	TBS2-teacher-sita.als
COMNT	
DATIM	Wed Dec 06 14:40:24 2017
OBNUC	1H
EXMOD	NON
OBFRQ	399.65 MHz
OBSET	124.00 KHz
OBFIN	10500.00 Hz
POINT	16384
FREQU	7992.01 Hz
SCANS	16
ACQTM	2.0500 sec
PD	4.9500 sec
PW1	6.00 usec
IRNUC	1H
CTEMP	16.9 c
SLVNT	CDCL3
EXREF	7.26 ppm
BF	0.10 Hz
RGAIN	20

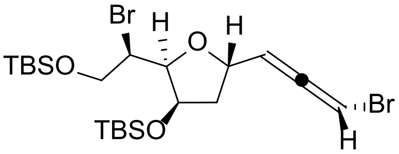

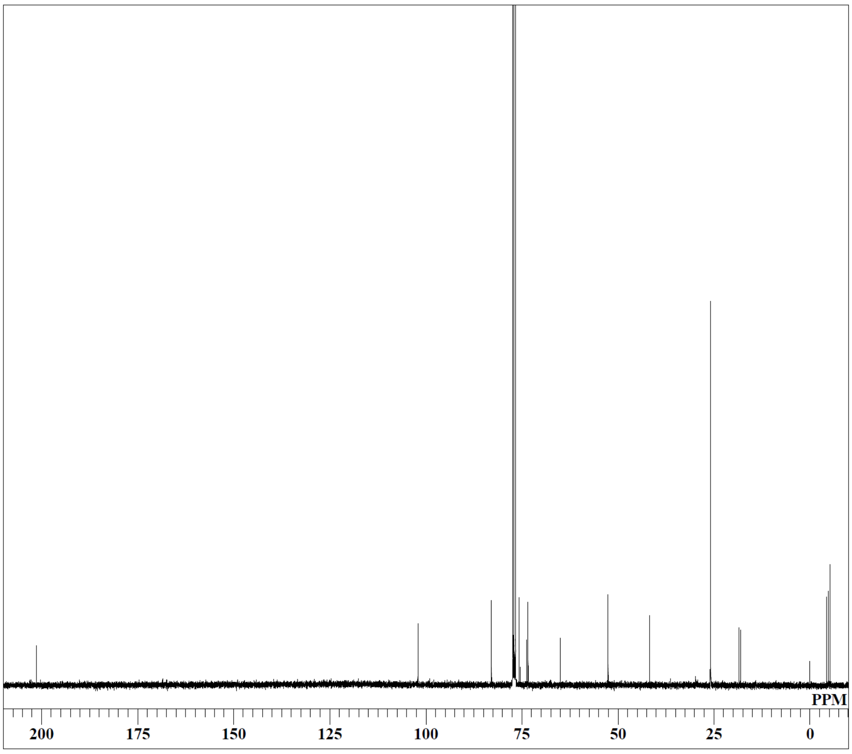

DFILE	TBS2-teacher-sita-13C.als
COMNT	auto
DATIM	Fri Feb 09 07:50:00 2018
OBNUC	13C
EXMOD	BCM
OBFRQ	100.40 MHz
OBSET	125.00 KHz
OBFIN	10500.00 Hz
POINT	32768
FREQU	27118.64 Hz
SCANS	14000
ACQTM	1.2083 sec
PD	1.7920 sec
PW1	5.00 usec
IRNUC	1H
CTEMP	20.8 c
SLVNT	CDCL3
EXREF	77.00 ppm
BF	0.10 Hz
RGAIN	31

(1*R*)-12

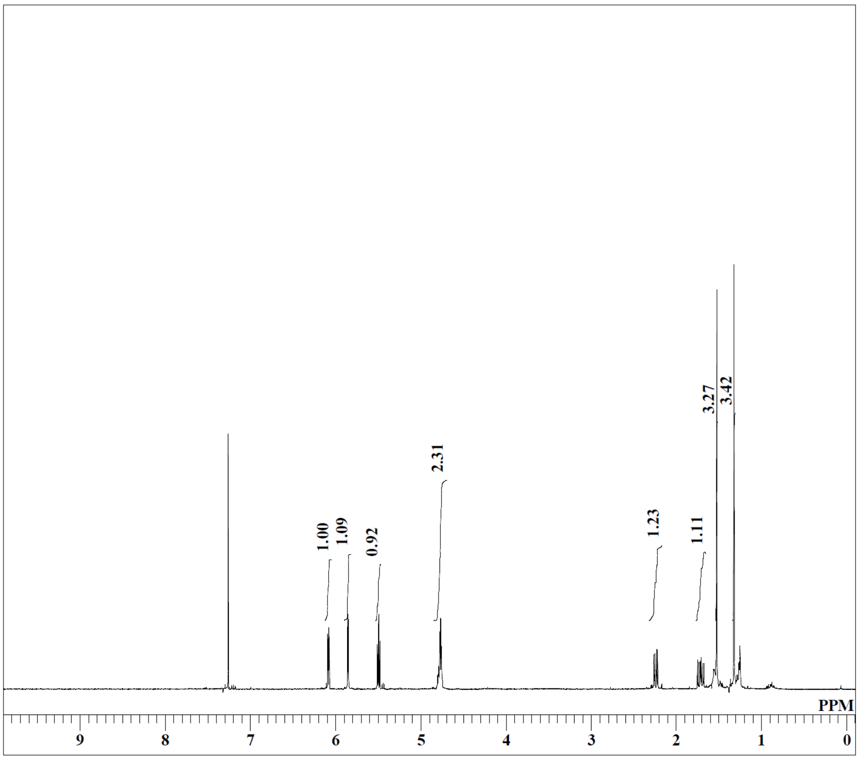

DFILE	no97-1-HPLC_study.als
COMNT	
DATIM	Fri Nov 17 20:18:39 2017
OBNUC	1H
EXMOD	NON
OBFRQ	399.65 MHz
OBSET	124.00 KHz
OBFIN	10500.00 Hz
POINT	16384
FREQU	7992.01 Hz
SCANS	8
ACQTM	2.0500 sec
PD	4.9500 sec
PW1	6.00 usec
IRNUC	1H
CTEMP	22.8 c
SLVNT	CDCL3
EXREF	7.26 ppm
BF	0.10 Hz
RGAIN	23

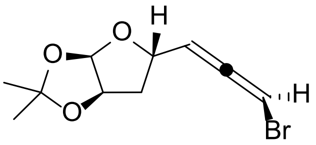

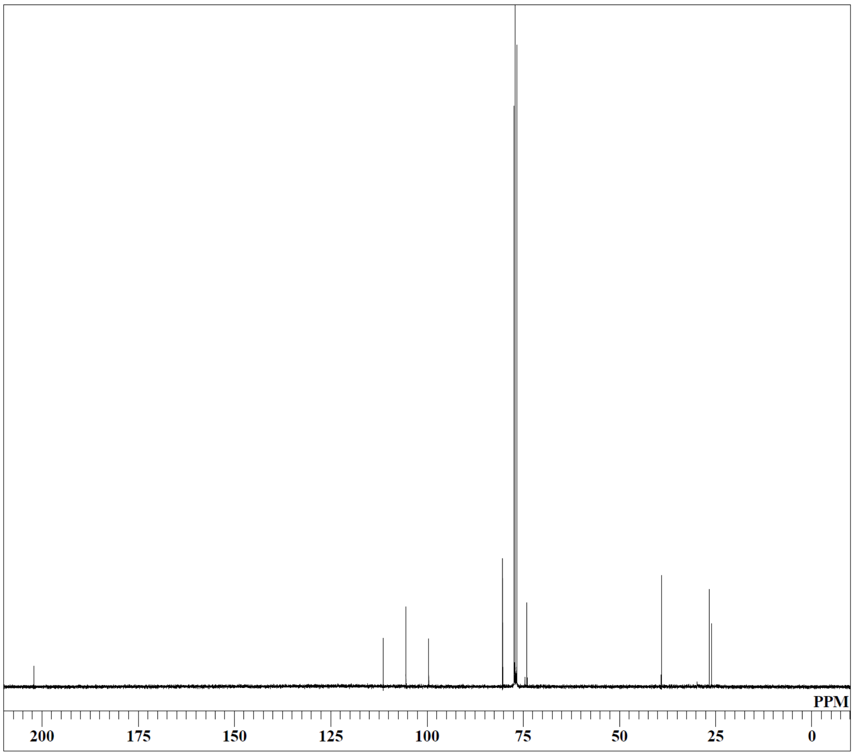

DFILE	no97-1-13C.als
COMNT	auto
DATIM	Sun Nov 19 07:55:59 2017
OBNUC	13C
EXMOD	BCM
OBFRQ	100.40 MHz
OBSET	125.00 KHz
OBFIN	10500.00 Hz
POINT	32768
FREQU	27118.64 Hz
SCANS	16400
ACQTM	1.2083 sec
PD	1.7920 sec
PW1	5.00 usec
IRNUC	1H
CTEMP	21.6 c
SLVNT	CDCL3
EXREF	77.00 ppm
BF	0.10 Hz
RGAIN	32

(1*R*)-13

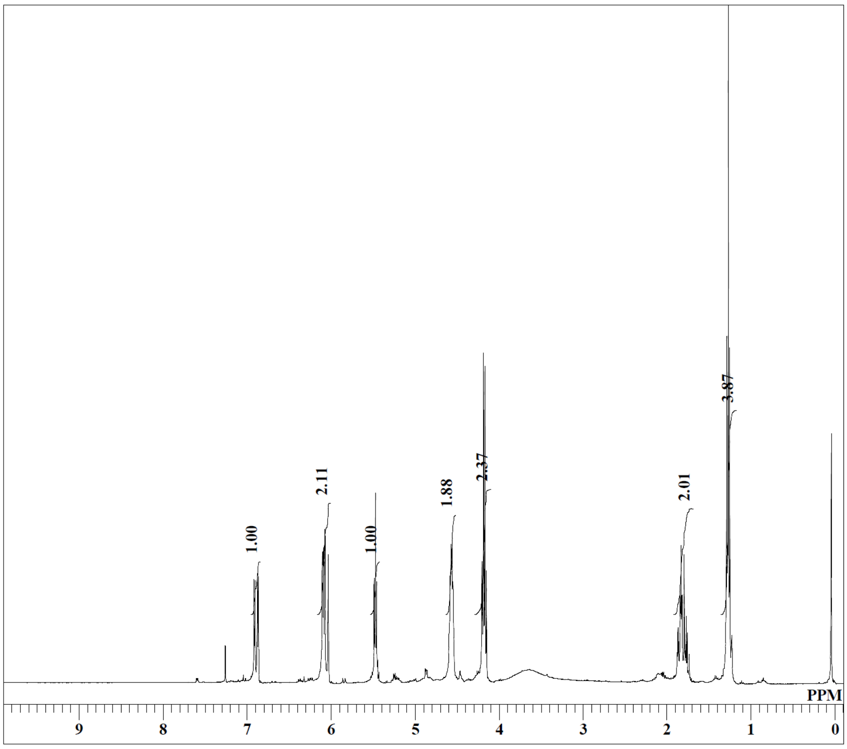

DFILE no51-1H.als
COMNT
DATIM Wed Aug 02 19:50:30 2017
OBNUC 1H
EXMOD NON
OBFRQ 399.65 MHz
OBSET 124.00 KHz
OBFIN 10500.00 Hz
POINT 16384
FREQU 7992.01 Hz
SCANS 16
ACQTM 2.0500 sec
PD 4.9500 sec
PW1 6.00 usec
IRNUC 1H
CTEMP 23.5 c
SLVNT CDCL3
EXREF 7.26 ppm
BF 0.10 Hz
RGAIN 14

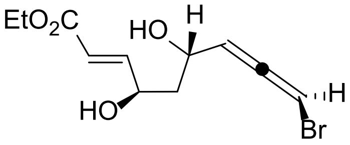

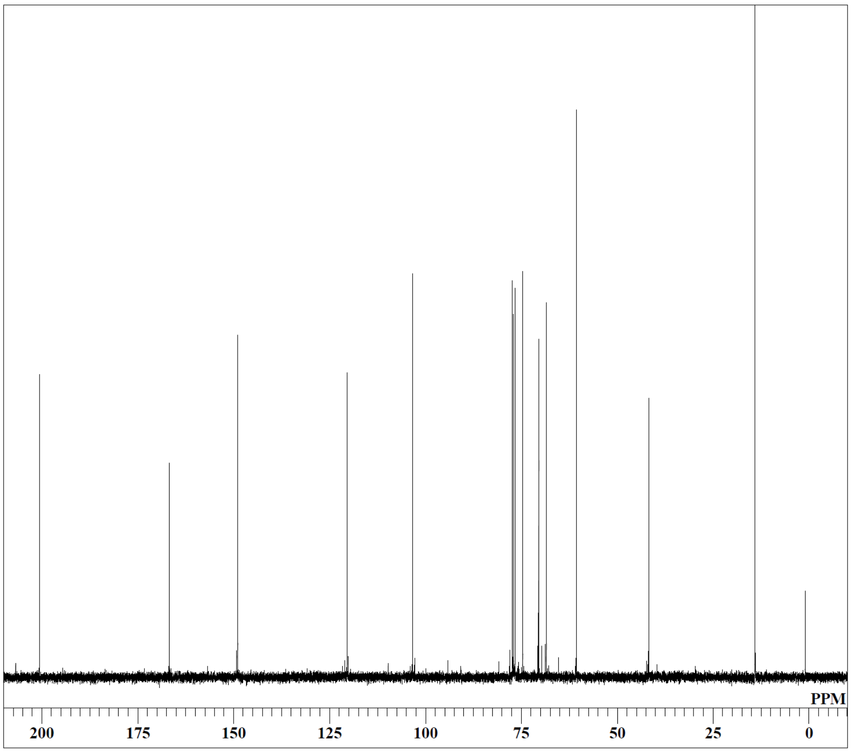

DFILE no51-13C.als
COMNT auto
DATIM Wed Aug 02 20:23:16 2017
OBNUC 13C
EXMOD BCM
OBFRQ 100.40 MHz
OBSET 125.00 KHz
OBFIN 10500.00 Hz
POINT 32768
FREQU 27118.64 Hz
SCANS 600
ACQTM 1.2083 sec
PD 1.7920 sec
PW1 5.00 usec
IRNUC 1H
CTEMP 23.0 c
SLVNT CDCL3
EXREF 77.00 ppm
BF 0.10 Hz
RGAIN 31

(1*R*)-14

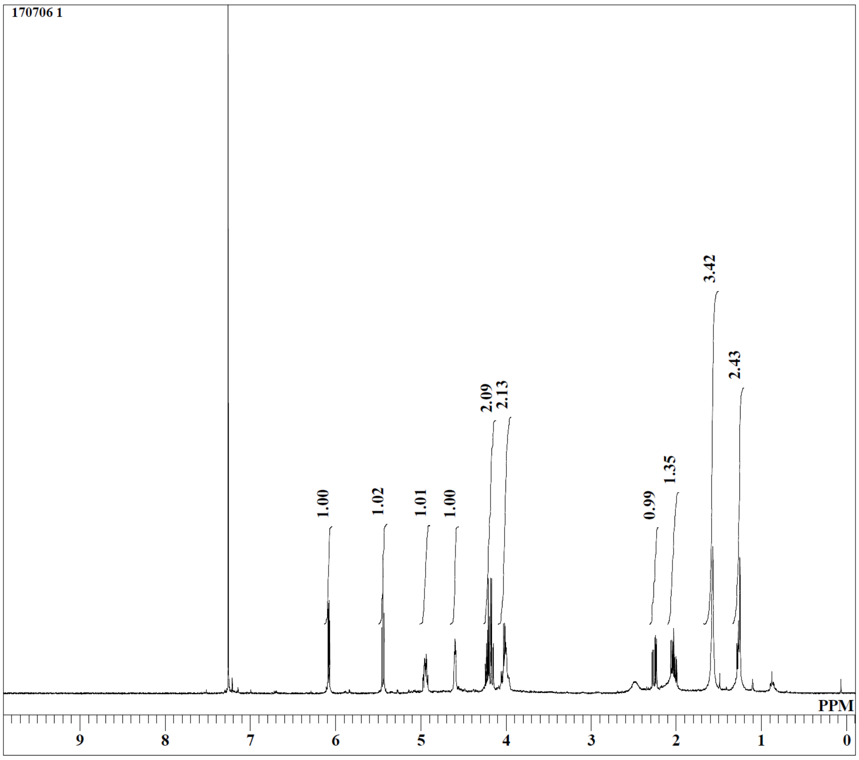

DFILE	no42-column2.als
COMNT	170706 1
DATIM	Thu Jul 06 16:08:16 2017
OBNUC	1H
EXMOD	NON
OBFRQ	399.65 MHz
OBSET	124.00 KHz
OBFIN	10500.00 Hz
POINT	16384
FREQU	7992.01 Hz
SCANS	8
ACQTM	2.0500 sec
PD	4.9500 sec
PW1	6.60 usec
IRNUC	1H
CTEMP	23.5 c
SLVNT	CDCL3
EXREF	7.26 ppm
BF	0.20 Hz
RGAIN	23

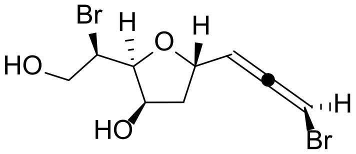

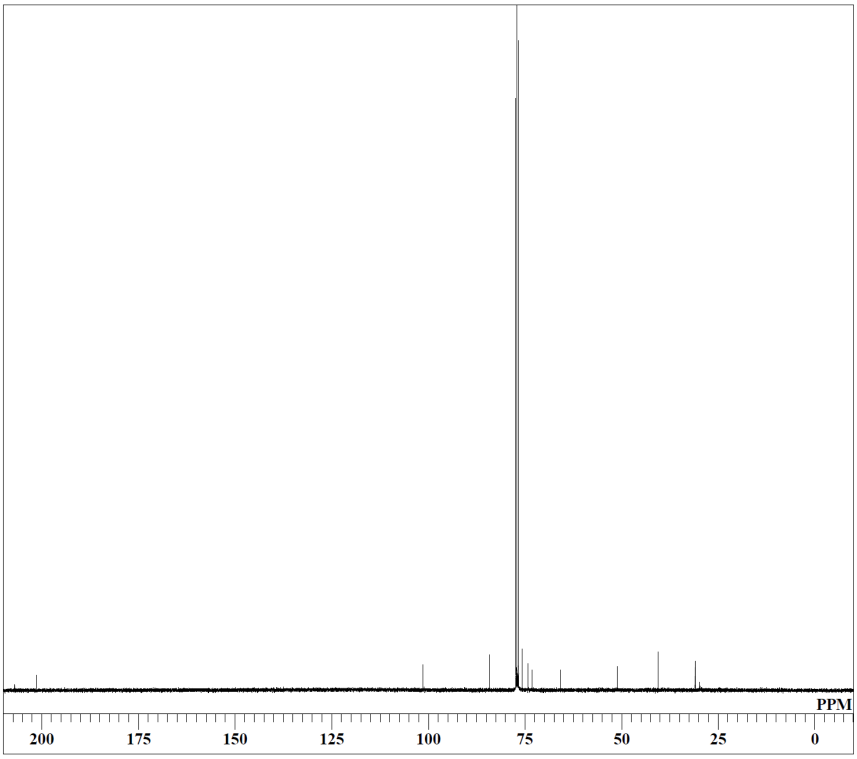

DFILE	No.42-2.als
COMNT	auto
DATIM	Sat Jul 08 08:10:46 2017
OBNUC	13C
EXMOD	BCM
OBFRQ	100.40 MHz
OBSET	125.00 KHz
OBFIN	10500.00 Hz
POINT	32768
FREQU	27118.64 Hz
SCANS	14000
ACQTM	1.2083 sec
PD	1.7920 sec
PW1	5.00 usec
IRNUC	1H
CTEMP	22.7 c
SLVNT	CDCL3
EXREF	77.00 ppm
BF	0.10 Hz
RGAIN	32

(1*R*)-15

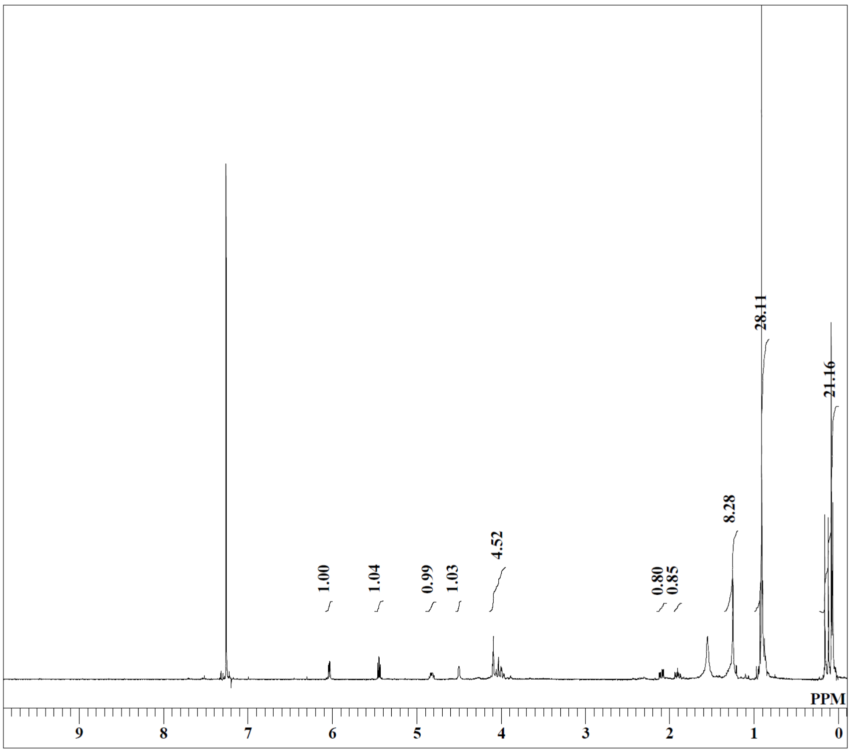

DFILE	no83-1-1H.als
COMNT	No.66 THF column C
DATIM	Thu Nov 23 17:14:09 2017
OBNUC	1H
EXMOD	NON
OBFRQ	399.65 MHz
OBSET	124.00 KHz
OBFIN	10500.00 Hz
POINT	16384
FREQU	7992.01 Hz
SCANS	16
ACQTM	2.0500 sec
PD	4.9500 sec
PW1	6.00 usec
IRNUC	1H
CTEMP	21.3 c
SLVNT	CDCL3
EXREF	7.26 ppm
BF	0.10 Hz
RGAIN	24

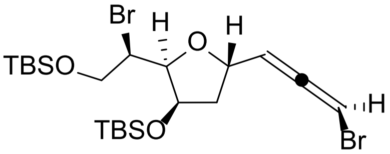

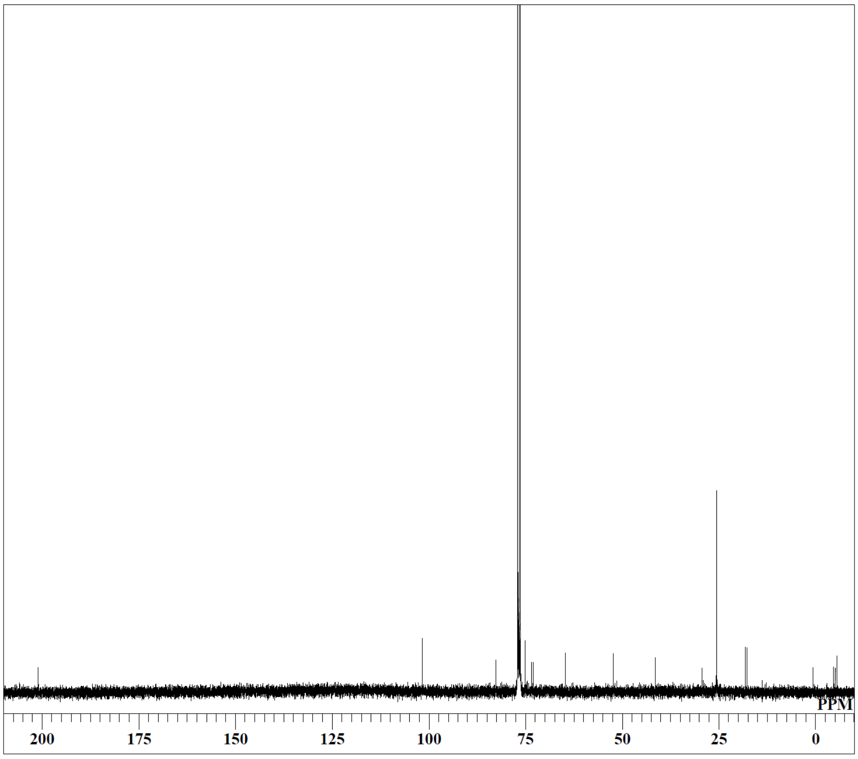

DFILE	no83-1-13C.als
COMNT	auto
DATIM	Fri Nov 24 06:57:06 2017
OBNUC	13C
EXMOD	BCM
OBFRQ	100.40 MHz
OBSET	125.00 KHz
OBFIN	10500.00 Hz
POINT	32768
FREQU	27118.64 Hz
SCANS	16400
ACQTM	1.2083 sec
PD	1.7920 sec
PW1	5.00 usec
IRNUC	1H
CTEMP	19.8 c
SLVNT	CDCL3
EXREF	77.00 ppm
BF	0.10 Hz
RGAIN	31

(1*R*)-8

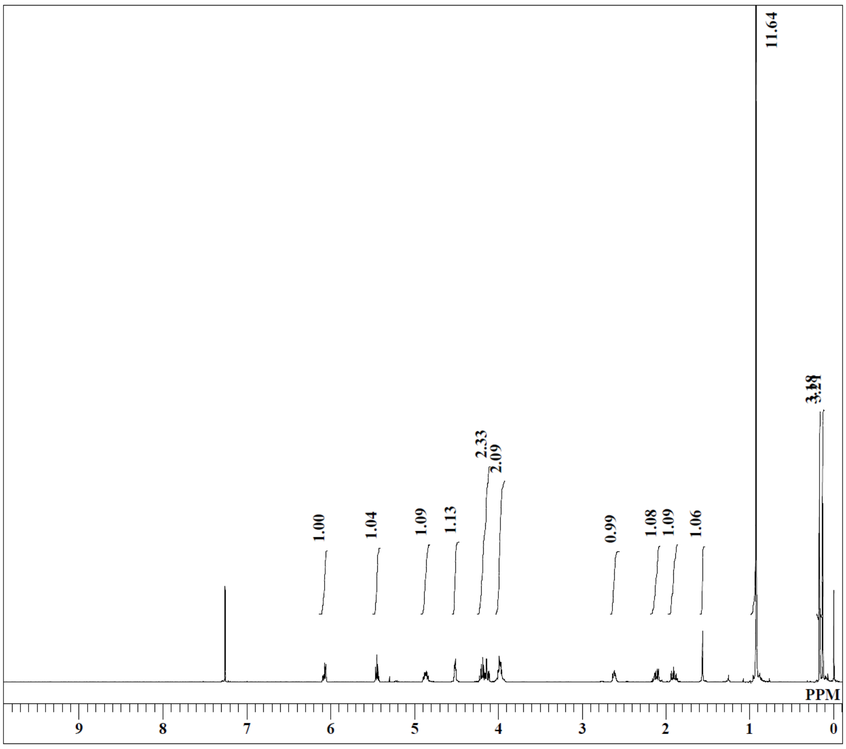

DFILE	no236-1H.als
COMNT	No.66 THF column C
DATIM	Mon Dec 03 19:36:13 2018
OBNUC	1H
EXMOD	NON
OBFRQ	399.65 MHz
OBSET	124.00 KHz
OBFIN	10500.00 Hz
POINT	16384
FREQU	7992.01 Hz
SCANS	16
ACQTM	2.0500 sec
PD	4.9500 sec
PW1	6.00 usec
IRNUC	1H
CTEMP	21.7 c
SLVNT	CDCL3
EXREF	7.26 ppm
BF	0.12 Hz
RGAIN	21

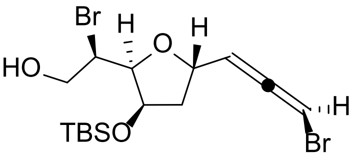

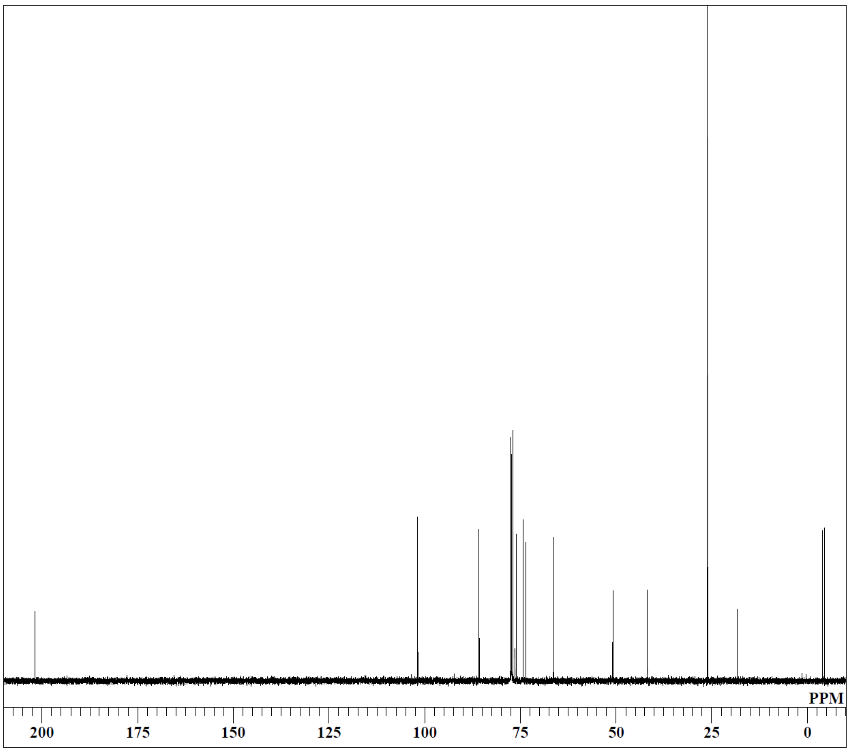

DFILE	no236-13C.als
COMNT	auto
DATIM	Tue Dec 04 20:55:12 2018
OBNUC	13C
EXMOD	BCM
OBFRQ	100.40 MHz
OBSET	125.00 KHz
OBFIN	10500.00 Hz
POINT	32768
FREQU	27118.64 Hz
SCANS	600
ACQTM	1.2083 sec
PD	1.7920 sec
PW1	5.00 usec
IRNUC	1H
CTEMP	22.6 c
SLVNT	CDCL3
EXREF	77.00 ppm
BF	0.12 Hz
RGAIN	32

MTPA Ester **A**

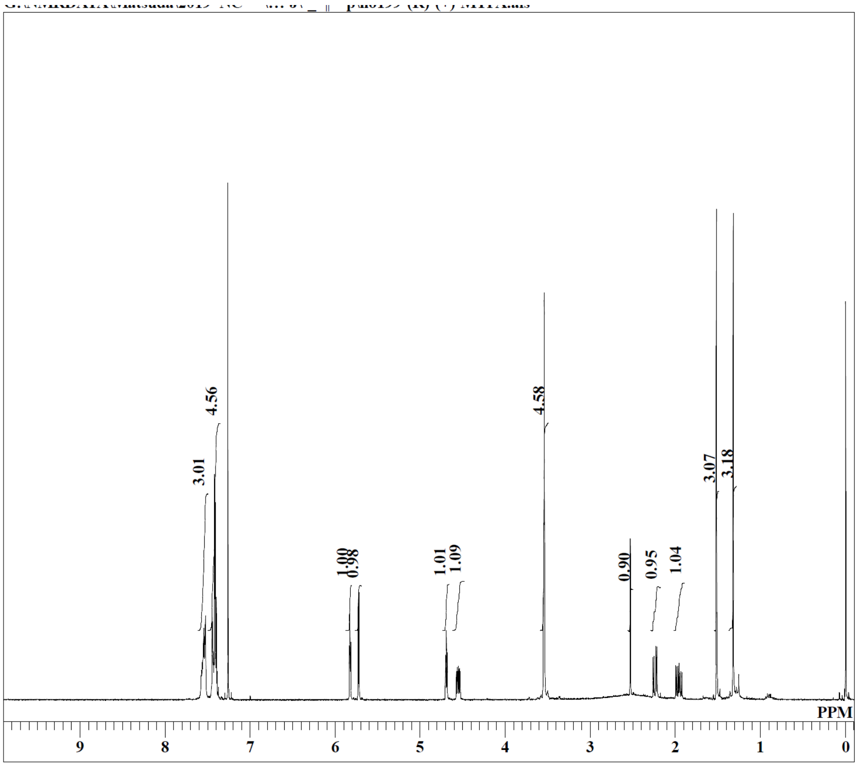

DFILE	no199-(R)-(+)-MTPA.als
COMNT	
DATIM	Sat Oct 13 13:50:22 2018
OBNUC	1H
EXMOD	NON
OBFRQ	399.65 MHz
OBSET	124.00 KHz
OBFIN	10500.00 Hz
POINT	16384
FREQU	7992.01 Hz
SCANS	8
ACQTM	2.0500 sec
PD	4.9500 sec
PW1	6.00 usec
IRNUC	1H
CTEMP	22.3 c
SLVNT	CDCL3
EXREF	7.26 ppm
BF	0.10 Hz
RGAIN	23

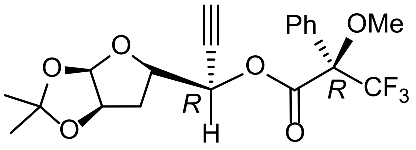

A

MTPA Ester **B**

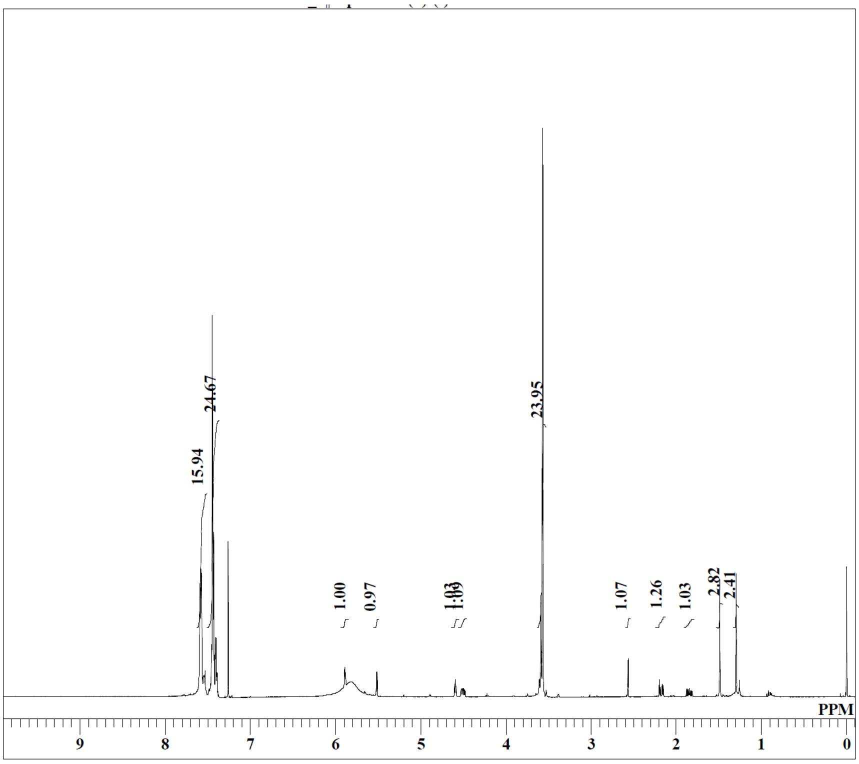

DFILE	no200-(S)-(-)-MTPA.als
COMNT	
DATIM	Sat Oct 13 13:59:27 2018
OBNUC	1H
EXMOD	NON
OBFRQ	399.65 MHz
OBSET	124.00 KHz
OBFIN	10500.00 Hz
POINT	16384
FREQU	7992.01 Hz
SCANS	16
ACQTM	2.0500 sec
PD	4.9500 sec
PW1	6.00 usec
IRNUC	1H
CTEMP	22.0 c
SLVNT	CDCL3
EXREF	7.26 ppm
BF	0.10 Hz
RGAIN	19

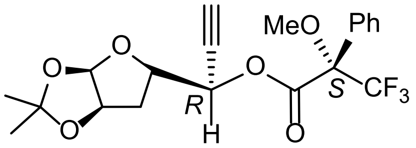

B

MTPA Ester **C**

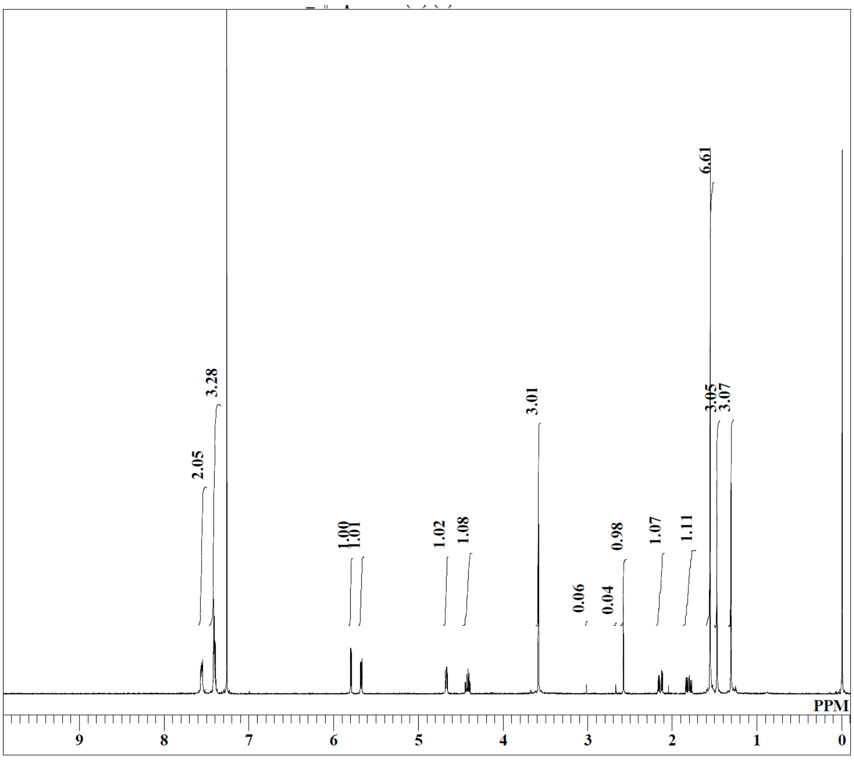

DFILE	no202-(R)-(+)-MTPA.als
COMNT	
DATIM	Sat Oct 13 11:02:35 2018
OBNUC	1H
EXMOD	NON
OBFRQ	399.65 MHz
OBSET	124.00 KHz
OBFIN	10500.00 Hz
POINT	16384
FREQU	7992.01 Hz
SCANS	8
ACQTM	2.0500 sec
PD	4.9500 sec
PW1	6.00 usec
IRNUC	1H
CTEMP	22.0 c
SLVNT	CDCL3
EXREF	7.26 ppm
BF	0.10 Hz
RGAIN	25

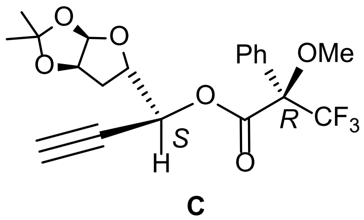

C

MTPA Ester **D**

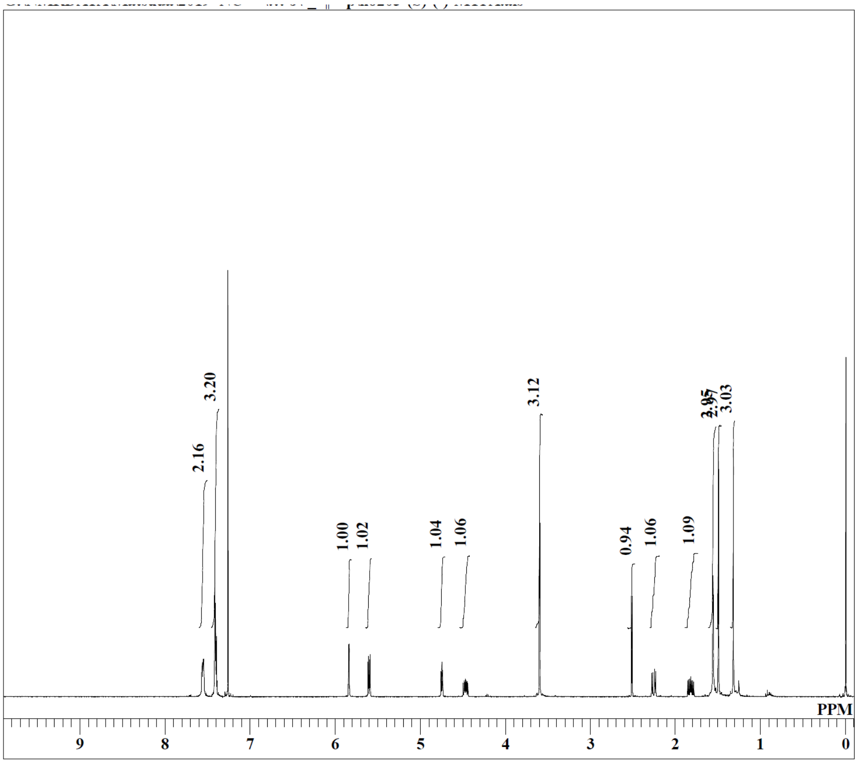

DFILE	no203-(S)-(-)-MTPA.als
COMNT	
DATIM	Sat Oct 13 11:10:37 2018
OBNUC	1H
EXMOD	NON
OBFRQ	399.65 MHz
OBSET	124.00 KHz
OBFIN	10500.00 Hz
POINT	16384
FREQU	7992.01 Hz
SCANS	8
ACQTM	2.0500 sec
PD	4.9500 sec
PW1	6.00 usec
IRNUC	1H
CTEMP	22.4 c
SLVNT	CDCL3
EXREF	7.26 ppm
BF	0.10 Hz
RGAIN	24

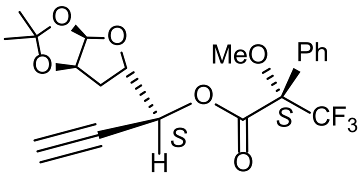

D
